# CD34 positive cells as endothelial progenitor cells in biology and medicine

**DOI:** 10.3389/fcell.2023.1128134

**Published:** 2023-04-17

**Authors:** Mehdi Hassanpour, Amankeldi A. Salybekov, Shuzo Kobayashi, Takayuki Asahara

**Affiliations:** ^1^ Shonan Research Institute of Innovative Medicine, Shonan Kamakura General Hospital, Kamakura, Kanagawa, Japan; ^2^ Center for Cell Therapy and Regenerative Medicine, Shonan Kamakura General Hospital, Kamakura, Kanagawa, Japan; ^3^ Kidney Disease and Transplant Center, Shonan Kamakura General Hospital, Kamakura, Kanagawa, Japan

**Keywords:** CD34 positive cells, endothelial progenitor cells, vasculogenesis, exosome, regenerative medicine

## Abstract

CD34 is a cell surface antigen expressed in numerous stem/progenitor cells including hematopoietic stem cells (HSCs) and endothelial progenitor cells (EPCs), which are known to be rich sources of EPCs. Therefore, regenerative therapy using CD34^+^ cells has attracted interest for application in patients with various vascular, ischemic, and inflammatory diseases. CD34^+^ cells have recently been reported to improve therapeutic angiogenesis in a variety of diseases. Mechanistically, CD34^+^ cells are involved in both direct incorporation into the expanding vasculature and paracrine activity through angiogenesis, anti-inflammatory, immunomodulatory, and anti-apoptosis/fibrosis roles, which support the developing microvasculature. Preclinical, pilot, and clinical trials have well documented a track record of safety, practicality, and validity of CD34^+^ cell therapy in various diseases. However, the clinical application of CD34^+^ cell therapy has triggered scientific debates and controversies in last decade. This review covers all preexisting scientific literature and prepares an overview of the comprehensive biology of CD34^+^ cells as well as the preclinical/clinical details of CD34^+^ cell therapy for regenerative medicine.

## 1 Introduction

**FIGURE 1:**
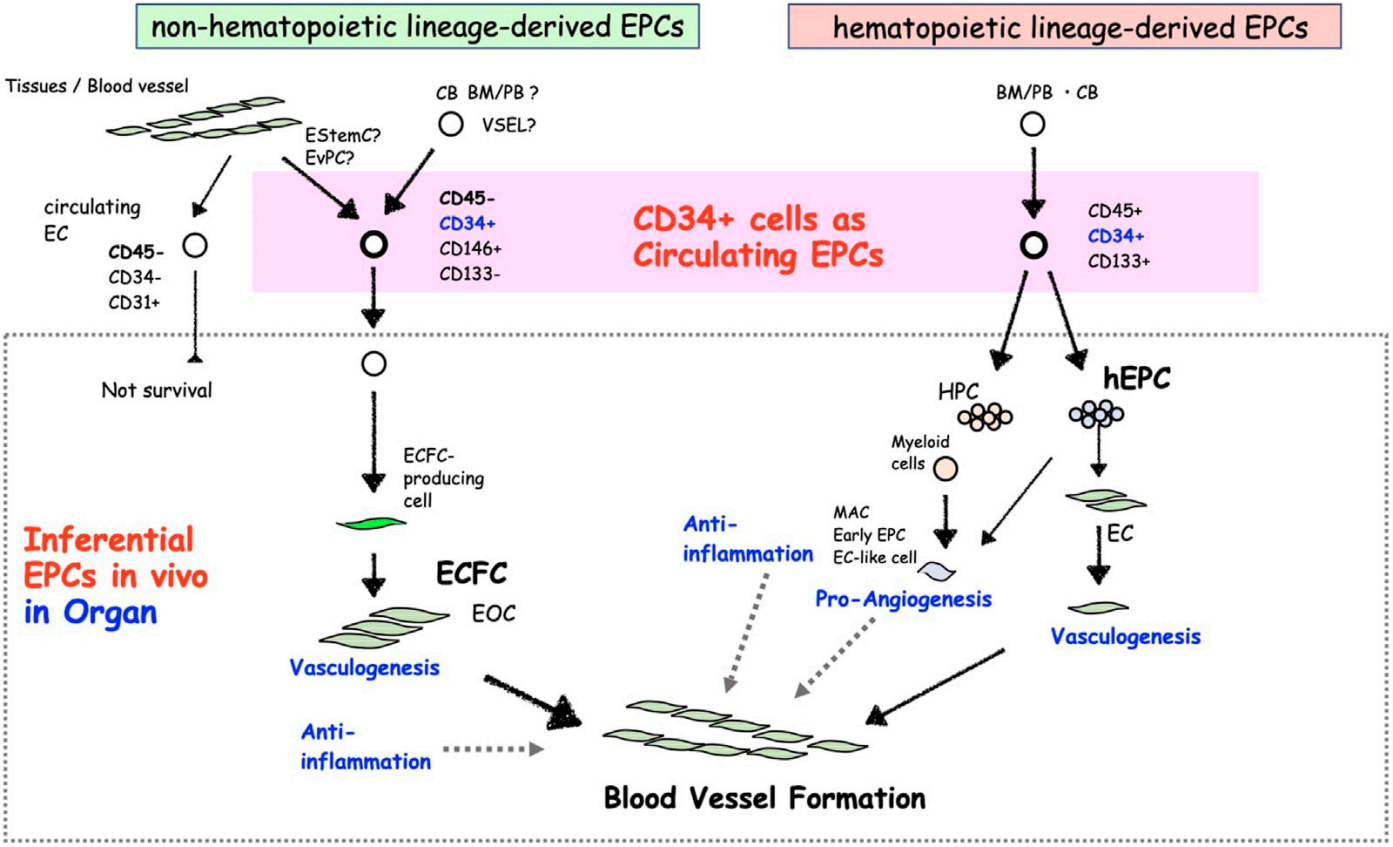
The concept of different CD34^+^ EPC types and their characteristics.

The CD34 surface marker is a sialomucin transmembrane protein composed of the haematopoietic progenitor cells (HPCs) antigen, podocalyxin, and endoglycan, which is a marker of vascular endothelial cells (ECs), HPCs, and endothelial progenitor cells (EPC) (Nielsen and McNagny, 2008). Definitely, using this cell marker allowed phenotyping and separation of circulating EPCs, which incorporate into ischemic areas and facilitate vasculogenesis (Asahara et al., 1997). Following the recognition of EPCs in peripheral blood (PB)-derived CD34^+^ cells (Asahara et al., 1997; Shi et al., 1998), EPC biology has been developed for years. Based on the initial hypothesis of hemangioblast, a natural origin of hematopoietic and endothelial lineages in embryogenesis, the identification of EPCs used cell surface markers of hematopoietic stem cells (HSCs) or HPCs, such as CD34, CD133, VEGFR-2, CXCR4, CD105, *etc.,* in human (Risau et al., 1988; Pardanaud et al., 1989; Flamme and Risau, 1992). Most of early research phase exploited CD34^+^ or CD133^+^ cell in human bone marrow (BM), PB or cord blood (CB) mononuclear cells (MNCs) and showed its commitment into endothelial lineage *in vitro* and its incorporation into ECs in neoangiogenesis *in vivo* (Murohara et al., 2000a; Gehling et al., 2000; Peichev et al., 2000; Quirici et al., 2001a; Kocher et al., 2001; Friedrich et al., 2006). These outcomes were backed up by the indisputable human clinical reports, indicating the incorporation of cells derived from CD34^+^ cells into the human vascular networks, in aortas of radiation syndrome patients (Suzuki et al., 2003), skin and gut from hematologic malignancy recipients (Jiang et al., 2004) and various cancer patients (Peters et al., 2005). Nevertheless, more than 2 decades of advances from the initial identification of EPCs, CD34^+^ or CD133^+^ cells are still pivoted as representative EPC-enriched cells for scientific EPC biology studies and therapeutical applications in clinical researches. As discussed later, the populations in circulation unintentionally comprehend both hematopoietic and non-hematopoietic lineage cell-derived EPCs (Figure 1).

**FIGURE 2:**
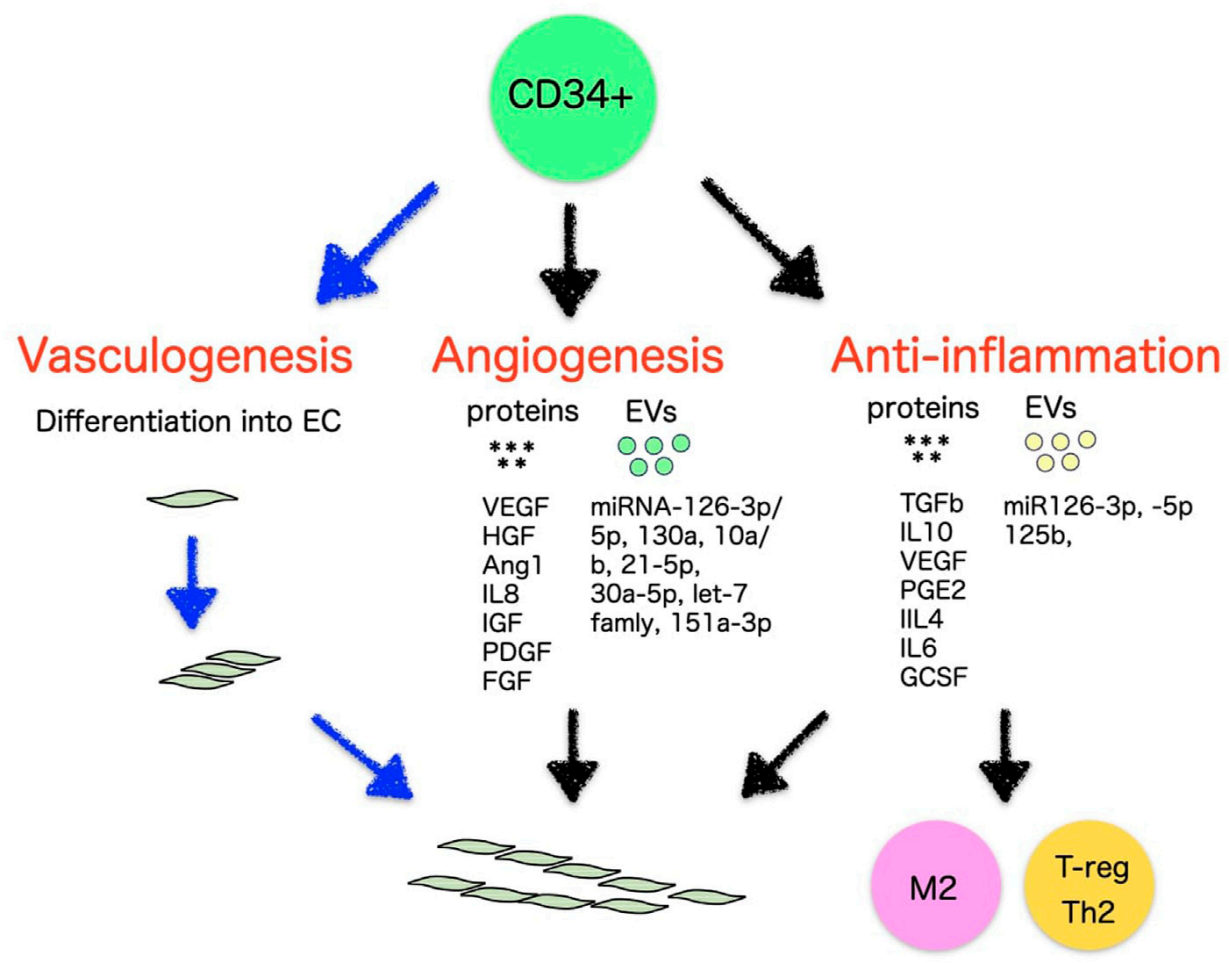
Vasculogenic, angiogenic, and anti-inflammatory mechanisms of CD34^+^ cells.

CD34^+^ populations have been the subject of far-reaching investigations and have been believed to have potential applications in regenerative therapy because of their unique regenerative properties (Velagapudi et al., 2019). In addition to the safety, feasibility, and potential efficacy of CD34^+^ EPC therapy demonstrated in the previous outcomes, as EPCs, CD34^+^ cells naturally have the capacity for self-renewal, making them a highly valuable source of stem cells in clinical settings and suggesting an important role for EPCs in therapeutic neovascularization. This may provide a reasonable justification for transplanting purified CD34^+^ EPCs in garden variety of disease. Based on the scientific and medical progress in recent EPC biology, CD34^+^ cells are reviewed in terms of progenitor biology for vasculogenesis, paracrine functions for angiogenesis and anti-inflammation, potency to differentiate into ECs or transdifferentiate, and therapeutic development in ischemic and inflammatory diseases (Figure 2).

## 2 CD34^+^ cell as a progenitor for ECs

The fact that the phenotype and function of EPCs must be discussed in terms of their cultured phenotype, “EPCs *ex vivo*,” has been viewed as an issue in the previous studies (Figure 2). Biologically, primary immature cells differentiating into ECs in the living organism should be referred as EPCs. However, many discussions have come to the conclusion that EPC biology is based on studies on differentiated cells into ECs in culture *ex vivo*, as is unavoidable in the progress of biological research. We must consider whether these debates about EPCs *ex vivo* are eventually aimed at “EPCs *in vivo*” cell biology. In this respect, we explain EPC biology in terms of its *in vivo* origin. One can distinguish two types of circulating EPCs *in vivo*, hematopoietic lineage cell-derived EPCs (hEPCs) and non-hematopoietic lineage cells-derived EPCs (non-hEPCs) (Asahara et al., 2011). hEPCs are cells derived from HSCs or HPCs mainly derived from the BM and upon stimulation capable of circulating as blood cells, represented for example by colony-forming (CF)-EPCs, non-CF-EPCs as differentiating EPCs in short-term culture, myeloid EPCs, lymphatic EPCs, angiogenic cells, *etc.* Non-hEPCs are neither likely derived from HSCs nor HPCs and can be isolated as EC phenotype *ex vivo* in adhesive long-term cell cultures from blood or tissue samples. Non-hEPCs supposed to mobilize from resident EPCs in organ blood vessels. Resident EPCs are considered as endothelial colony forming cell (ECFC)-producing cells (Patel et al., 2013a; Lin et al., 2013; Alphonse et al., 2014; Green et al., 2017a), and have been identified in mice such as endothelial stem cells or endovascular progenitors (Patel et al., 2017; Wakabayashi et al., 2018), despite the fact that this has not yet been defined in human terms.

### 2.1 CD34^+^ cells as non-hEPCs

Non-hEPC is almost unified to one kind of endothelial outgrowth cells (EOCs). EOCs-produced from PB *ex vivo* reported initially by Lin et al. (2000). The concept of adhesive endothelial lineage cell stages using the original culture conditions disclosed clonal colony-forming units of outgrowth ECs cultured from MNCs or blood vessel ECs by Ingram and Yoder *et al.* (Ingram et al., 2004; Ingram et al., 2005a). These proliferative endothelial lineage cells were termed ECFC, also known in the literature as EOCs or late EPCs. ECFCs do not likely represent the primary “EPC *in vivo*” phenotype found in our body but rather reflect an EC phenotype emerging from cultured cells (Lin et al., 2000) or vessel wall ECs (Figure 1) (Ingram et al., 2005b). Questions regarding the definition of the primary EPC underlying the ECFC phenotype and *in vivo* origin are still missing. Initially, ECFCs were identified from the long-term culture of PB (Lin et al., 2000; Hur et al., 2004) or CB-MNCs (Ingram et al., 2004). These were followed by publication of Smadja *et al.* and Delorme *et al.*, showing ECFCs can be derived from CD34^+^ cells or CD146^+^/CD34^+^/CD45^+^/CD133^+^ or CD177^+^ cells (Delorme et al., 2005; Smadja et al., 2005; Lee et al., 2013; Boisson-Vidal et al., 2018). Additional studies by Case *et al.* and Timmermans *et al.* revealed that CD34^+^/CD45^−^ cells could give rise to ECFCs but not a hematopoietic colony (Case et al., 2007; Timmermans et al., 2007). A later publication by Tura et al. confirmed CD34^+^/CD146^+^/CD133^−^ cells are the source of ECFC precursor cells (Tura et al., 2013). They also claimed ECFC precursors might not be derived from BM, because BM-MNCs did not contain CD34^+^/CD146^+^/CD133^−^ cells and did not give rise to any ECFC colony. This discussion was partly supported by a recent publication using fluorescence *in situ* hybridization analysis of ECFCs derived from allogenic BM transplant patients (Fujisawa et al., 2019). The findings about the fraction of ECFC-producing cells *in vivo* are somewhat contradicting between the publications regarding CD45 positivity and CD133 positivity, while CD34 positivity is agreed by common consent.

ECFCs have been successfully isolated from not only CB and PB but also from fat tissue (Lin et al., 2013), placenta (Patel et al., 2013b), lungs (Alphonse et al., 2014) and saphenous vein (Green et al., 2017b); these results imply that ECFCs may derived from tissue resident vasculature progenitors. Recent reports indicate a candidate of ECFC origin locates in ECs of blood vessels (Salybekov et al., 2022). The mechanism of non-hEPC mobilization into circulation and physiological functions in kinetics are not defined yet.

Although the proof of BM-derived ECFCs is not approved by the former reports, there are several publication demonstrating that a pluripotent stem cell known as “very small embryonic-like stem cell” (VSEL) is a lineage-negative, CD34^+^, CD133^+^ and CD45^−^ cell with very small size (<6 µm length) located in BM, and differentiate into ECFC-like ECs in human (Havens et al., 2014; Guerin et al., 2015; Lahlil et al., 2018; Domingues et al., 2022). Also other reports disclosed VSELs derived from cord blood CD34^+^ cell differentiated into ECFCs *ex vivo* (Lahlil et al., 2018) (Domingues et al., 2022). We need further insights and discussions regarding the origin of ECFCs.

### 2.2 CD34^+^ cells as hEPCs

Postnatal EPCs was thought closely related to that of HSC populations, both being isolated by cell surface antigens, including CD34, CD133, VEGFR-2, CXCR4, CD105, *etc.,* in human, or c-Kit, Sca-1, Flk-1, *etc.,* in mouse (Peichev et al., 2000; Quirici et al., 2001b; Eggermann et al., 2003; Handgretinger et al., 2003; Alessandri et al., 2004). However, the identification of an exact primary hEPC phenotype was missing, mainly due to the lack of a definitive assay system capable of determining and distinguishing an EPC unambiguously from an HSC.

The initially developed culture assays and colonies (Murohara et al., 2000b; Kalka et al., 2000; Hill et al., 2003) grouped multifarious EPCs into single qualitative class: “adhesive cultured EPCs”, without any hierarchy or proper identification of unwanted cells, while the colonies detected by Hill’s colony assay (Hill et al., 2003) turned out to consist of not colony-forming EPCs, but mainly other hematopoietic cells (Hur et al., 2007; Rohde et al., 2007). Later, an improved EPC-colony forming assay (EPC-CFA) was developed, challenging the field’s traditional views and opening the door to illustrating the developmental hierarchy of hEPCs (Masuda et al., 2011).

#### 2.2.1 Colony forming hEPCs

The EPC-CFA enables the description and discrimination of two different forms of EPC-CFUs derived from a single cell; small cell-sized EPCs as “primitive CF-EPCs”, which are probably derived from more immature/proliferative EPCs, and large cell-sized EPCs as “definitive CF-EPCs”, which have more tendency to differentiation and functional participation in neovasculogenesis-related process (Table 1). The definitive CF-EPCs are more talented to differentiate into a non-colonizing, large cell-sized EPC phenotype, parallel to classical EPC culture-derived EPCs (Dimmeler et al., 2001; Vasa et al., 2001) and are considered as newly emerging EPCs, leaving the colony-forming EPCs niche (Figure 1). Masuda *et al.* demonstrated that approximately 22.0% of a single CB-CD133^+^ gave rise to primitive or definitive EPC colonies (16.8% or 5.2%, respectively) in EPC-CFA. This assay can further be combined with an HPC colony assay to elucidate the rationale of a possible “bloodline” between EPCs and HPCs. EPC-CFA along with HPC-CFA, opens the door for additional visions into the origin and characteristics of hematopoiesis and neovasculogenesis in the adult (Masuda et al., 2011). An only CB-CD133^+^ cell could produce both EPC and HPC colonies at a rate of 36.3% after particular culture conditioning for 7 days.

**TABLE 1:** Phenotypes of CD34^+^ EPCs.

	Phenotype	Assay	Flow cytometry antigens	
			Positive	Negative
non-hematopoietic EPCs	ECFCs	*ex-vivo* identified in ECFC culture assay	CD34/CD31/CD144 (CD133^+/−^)	CD45/CD14/CD41a
hematopoietic EPCs	colony forming EPCs	identified by EPC-CFA assay	CD34/CD133	CD14
	Myeloid EPCs	EC culture differentiation from CMP or GMP	CD34/CD38/CD117/Tie2	CD10/CD135
	Lymphoid EPCs	VEGFR3/LYVE-1 expressing EC differentiation from myeloid cells	CD34/CD38/VEGF-R3/CD11b	

EPCs, Endothelial progenitor cells; ECFCs, Endothelial colony-forming cells.

#### 2.2.2 Myeloid and Lymphoid hEPCs

Several former publications claimed that myeloid early EPCs like myeloid angiogenic cells (MACs) and circulating angiogenic cells (CACs) should not be involved in the EPC category, because they were not committing into totally differentiated ECs but functioning as myeloid cells to deliver angiogenic mediators (Medina et al., 2017). As this partly agreeable, myeloid EPCs are not only MACs or CACs but also progenitor cells that differentiate into ECs of blood vessel in tumors and regenerative tissues. Bailey *et al.* described common myeloid progenitors (CMPs) and granulocyte/macrophage progenitors (GMPs) could differentiate into functional ECs by 1.3% and 0.8%, respectively (Bailey et al., 2004; Bailey et al., 2006), followed by Romangnani *et al.* showed that CD14^+^/CD34^low^ MNCs with stem cell phenotypic and functional features represent the major source of circulating EPCs with high vasculogenic property (Romagnani et al., 2005). Furthermore, the research flow of myeloid EPCs is extended to lymphatic EPCs, which are derived from myeloid cells. The concept of lymphatic vessel development has recently been revised by evidence rising from both mouse and human studies for the critical role of lymphatic EPCs (LEPCs) (Ran and Wilber, 2017; Ran and Volk-Draper, 2020). Adult LEPCs have been found to be derived from wide variety of ancestors, including HSC (Jiang et al., 2008), MSC (Conrad et al., 2009), ADSC (Yang et al., 2015), and myeloid stem or progenitor cells (Lee et al., 2010), with the myeloid lineage gaining popularity as the primary source (Maruyama et al., 2005; Zumsteg et al., 2009; Li et al., 2014). BM-derived immature myeloid cells recruit to inflamed and ischemic sites (Maruyama et al., 2005) and tumors (Allavena et al., 2008; Pollard, 2008), where they help to develop vasculature (Schoppmann et al., 2006; Ding et al., 2012). One of the compelling lines of evidence for myeloid EPCs is the ability to differentiate from primary blood monocytes under *in vitro* conditions, demonstrating *de novo* property of lymphatic phenotype expressing such as VEGFR3 and LYVE-1, endothelial-specific phenotype, and functional behaviors limited to vasculature cells (Van’t Hull et al., 2014; Tan et al., 2014), concurrent with the loss or reduction of stem, myeloid, and hematopoietic markers (Salven et al., 2003; Nguyen et al., 2009).

## 3 CD34^+^ cells as commanders for angiogenesis and anti-inflammation

### 3.1 EPC-derived angiogenic proteins

EPCs are thought to release autocrine elements for their nutritional support, as well as paracrine factors to develop vasculature and repair of impaired vessels (Figure 2). *In vivo* EPCs, isolated as CD34^+^ from PB or BM, have been reported to secrete numerous angiogenic elements, such as vascular endothelial growth factor (VEGF), hepatic growth factor (HGF), insulin growth factor-1 (IGF-1), interleukin-8 (IL-8), fibroblast growth factor (FGF) (Majka et al., 2001; Jarajapu et al., 2014) angiopoietin-1, platelet-derived growth factor (PDGF) (Fang et al., 2020), *etc. Ex vivo* EPCs such as early EPCs as well as ECFCs also represented a similar finding to express a large number of angiogenic factors although the level of VEGF and IL-8 were higher in early EPCs than ECFCs (Table 2) (Cheng et al., 2013).

**TABLE 2:** Angiogenesis and anti-inflammatory proteins secreted by EPCs.

Secreted proteins	Type of EPCs	Effects	Ref
VEGF	ECFCs	↓ inflammation	Jarajapu et al. (2014)
HGF	ECFCs	↓ inflammation	Majka et al. (2001), Jarajapu et al. (2014)
		↑ immunosuppression	
IGF-1	ECFCs	↑anti-inflammatory response	Majka et al. (2001)
Ang-1	ECFCs	↑ anti-inflammatory response	Majka et al. (2001)
PDGF	ECFCs	↓ inflammation	Fang et al. (2020)
		↑ immunosuppression	
FGF	ECFCs	↓ inflammation	Majka et al. (2001)
PGE2	ECFCs	↓platelets translocation, glycoprotein IIb/IIIa activation, aggregation, and adhesion to collagen	Carneiro et al. (2019)
IL-4	Colony forming EPCs	↑ immunosuppression and anti-inflammatory response	Kado et al. (2018)
IL-6	Colony forming EPCs	↑angiogenesis and immunosuppression	Kado et al. (2018)
IL-8	Myeloid EPCs	↑ angiogenic response	Cheng et al. (2013)
IL-10	ECFCs	↑ immunosuppression	Zullo et al. (2015)
		↑ anti-inflammatory response	
TGF-β	Colony forming EPCs	↓pro-inflammatory cytokine	Acosta et al. (2019)
		↑ anti-inflammatory response	
		↑immunosuppression	
M-CSF	ECFCs	↑ immunosuppression	Alwjwaj et al. (2021)
		↓inflammation	
G-CSF	Colony forming EPCs	↑ immunosuppression	Wen et al. (2019)
		↑ anti-inflammatory response	

### 3.2 EPC-derived anti-inflammatory proteins

Collaborative factors with the capacity to promote both immunomodulation and anti-inflammation, such as TGF-β, IL-10, IL-4, IL-6, PGE2, M-CSF, G-CSF, adenosine, *etc.*, have been investigated for years and reported to be expressed by properly stimulated hematopoietic lineage cells (Motz and Coukos, 2011; Rivera and Bergers, 2015). This co-creative property inherently applies to EPCs in regenerative tissues. EPCs secrete multiple factors for both anti-inflammation and immunosuppression, such as VEGF (Jarajapu et al., 2014), PGE2 (Carneiro et al., 2019), TGF-β (Acosta et al., 2019), IL-4, IL-6 (Kado et al., 2018), IL-10 (Zullo et al., 2015) and G-CSF (Wen et al., 2019; Alwjwaj et al., 2021; Yan et al., 2022). Especially, IL-10 and TGF-β are expressed and play a key role in immunomodulation and anti-inflammation in CD34^+^ cells from PB or BM (Majka et al., 2001; Ratajczak et al., 2013; Jarajapu et al., 2014). IL-10 is a strong anti-inflammatory and immunoregulatory cytokine with a variety of direct and indirect effects on innate and adaptive immunity through the induction of M2 macrophages and Treg polarization (Mosser and Zhang, 2008). In acute myocardial infarction (MI) models, IL-10 suppresses *in vivo* inflammatory responses, which contributes to improved myocardial recovery. TGF-β_1_ is a multifunctional cytokine involved in a variety of essential activities, such as embryonic evolution, cellular development, wound recovery, and immunomodulation, which contributes to the anti-inflammatory feedback by encouraging the proliferation of Tregs and inhibiting the differentiation of Th cells (Mantel and Schmidt-Weber, 2011).

### 3.3 EPC-derived miRNAs in extracellular vesicles

MicroRNAs (miRNAs) are a class of non-coding RNAs and have critical functions in the modification of gene expression. Most of them are transcribed from DNA into primary miRNAs, modified into initial miRNAs, and ultimately made into mature miRNAs (Mohr and Mott, 2015). Seminal study showed that CD34^+^ EPC secreted extracellular vesicles (EVs) and activated an angiogenic program in ECs through horizontal mRNA transfer (Table 3) (Deregibus et al., 2007; Sahoo et al., 2011; Xu et al., 2020; Xiong et al., 2022).

**TABLE 3:** Angiogenesis and anti-inflammatory EV-miRs secreted by EPCs.

Secreted miRs	Type of EPCs	Mechanisms	Effects	Ref
miR-126 and miR-130a	ECFCs	↓*SPRED-1, VCAM, MCP1*	↑angiogenesis	Sahoo et al. (2011)
			↓inflammation and oxidative stress	
miR-221-3p	Colony forming EPCs	↑ *VEGF, CD31 Ki67*	↑angiogenesis	Xu et al. (2020)
		↓AGE-RAGE signaling pathway	↓inflammatory response	
miR-30d-5p	Colony forming EPCs	↑ M2 macrophages, CCL17, CCL22, VEGF, FGF21, NRF2, and HO-1	↑angiogenesis and immunosuppression	Xiong et al. (2022)
		↓ M1 macrophages, IL-1β and TNF-α	↓inflammatory response	
miR-126-3p	ECFCs	↓*SPRED-1*	↑angiogenesis	Mathiyalagan et al. (2017)
		↑*VEGF, ANG1, ANG2, MMP9, TSP1*		
10a/b, miR-21-5p, miR-30a-5p, miR-126-5p, let-7 families, miR151a-3p	ECFCs	↑*SERPINB, TPM2, FOXD1, MMP1, MIR 1974, TMEM200A, DSE, TMEM154, TPM2, FBLN2*	↑angiogenesis and anti-inflammation	Dellett et al. (2017)
		↓ *ALDH1A1, ITM2A, CRYAB, SULF1, AIF1L, CCL2, BGN, EDN, ACVRL1*		
miR-218-5p and miR-363-3p	ECFCs	↑ CD31, VEFGR2	↑angiogenesis and anti-inflammation	Ke et al. (2021)
		↓ Vimentin, α-SMA by targeting p53/JMY-mediated cell apoptosis and mesenchymal-endothelial transition		
miR-21-5p	ECFCs	↑syndecan-1	↑ anti-inflammatory response	Zhang et al. (2021)
		↓RUNX1, heparanase-1	↓ apoptosis and oxidative stress response	
miR-486-5p	ECFCs	↑Akt phosphorylation	↓inflammatory response	Viñas et al. (2016)
		↓ PTEN		

AGEs, advanced glycation end-products; RAGE, advanced glycation end-product receptor.

#### 3.3.1 Cardiovascular diseases

Cardiovascular diseases are based on pathological process of ischemia caused by circulatory insufficiency and inflammation induced by hematopoietic cell invasion, requiring angiogenic and anti-inflammatory microenvironment for tissue recovery. Preclinical studies have shown that EVs derived from CD34^+^ have superior therapeutic effects on various ischemic diseases (Sahoo et al., 2011; Bitzer et al., 2012; Mathiyalagan et al., 2017). Sahoo *et al.* have shown pro-angiogenic activity of EVs from conditioned media of mobilized human CD34^+^ cells (CD34^+^-EVs) possibly through miR-126 and 130a (Sahoo et al., 2011). According to Mathiyalagan and colleagues, silencing miR-126-3p in CD34^+^-EVs derived from G-CSF mobilized PB-MNCs eliminated their angiogenic behavior and other constructive activities *in vitro* as well as *in vivo.* In addition, infusion of CD34^+^-EVs improved miR-126-3p levels in mouse ischemic limbs while having no impact on endogenous miR-126-3p synthesis, implying a horizontal transfer of efficient miR-126-3p to the ischemia site (Mathiyalagan et al., 2017). Dellet *et al.* documented highly expressed miRs identified in EVs produced by CD31^+^/CD34^+^/CD146^+^ ECFC and ECFC obtained from umbilical cord blood (UCB)-MNCs, including miR-10a/b, miR-21-5p, miR-30a-5p, miR-126-5p, miR151a-3p, let-7 families, *etc.* (Dellett et al., 2017). However, EPC-EVs works for anti-inflammatory effect on microenvironment of ischemia tissues as well. Later, Venkat *et al.* reported that EVs derived from CD133^+^EPC administration increased miR-126 levels while decreasing in *MCP-1* and *VCAM1*, leading to decreased cardiac inflammation and oxidative stress after stroke in T2DM mice (Venkat et al., 2021). Also, BM-ECFC-derived EVs mitigate atherosclerosis, inflammation response, and atherosclerosis-related endothelial dysfunction in the experimental mice DM models (Bai et al., 2020). Ke *et al.* also reported that PB-EPC-EVs including miR-363-3p and miR-218-5p have cardioprotective effects on MI damage by targeting JMY-related apoptosis and mesenchymal-endothelial transition (Ke et al., 2021). Zhuo *et al.* realized that *Rab27a* deletion (as one of the major genes involved in EV biogenesis) weakened the therapeutic functionality of EPCs in MI conditions. Mechanistically, *Rab27a* deletion inhibits the PI3K/cyclinD1/Akt/FoxO3a pathway and reduces EV secretion in CD34^+^/VEGFR-2^+^ EPCs (Zhou et al., 2021). In turn, it is worth noting that suppression of PI3K/Akt induces inflammatory cascades (Schabbauer et al., 2004; Zhao et al., 2019). Therefore, it could be concluded that EV knockouts in EPCs initiate the inflammation process, which is another reason for the anti-inflammatory properties of intact EPCs for CVDs.

#### 3.3.2 Acute lung injury/acute respiratory distress syndrome

Acute lung injury/acute respiratory distress syndrome (ALI/ARDS) mortality rate is approximately 40% and mostly caused by sepsis, pneumonia, severe traumas, *etc.* (Dushianthan et al., 2011). Initial reports showed that BM derived CD31^+^/Flk1^+^/CD34^+^-EV infusion significantly decreased LPS-induced lung inflammatory process, representing convincing anti-inflammatory effects of these cells. The histologic analysis of CD34^+^-EVs injected group revealed restricted edema, neutrophil recruitment, and cytokines/chemokines level reduction in the bronchoalveolar lavage (BAL) (Wu et al., 2018). From a mechanistic viewpoint, miR-126 is abundant in CB-EPC-EVs, and upregulation of miR-126-3p may target PIK3R2, whereas upregulation of miR-126-5p prevents HMGB1 from acting as an inflammatory alarmin and VEGF- from acting as a permeability factor (Zhou et al., 2019). EPC-EVs has constructive functions to improve ALI/ARDS consequences, and additional investigations are mandatory to outline the best EV delivery method to damaged tissue.

#### 3.3.3 Sepsis

Sepsis is systemic inflammatory condition provoked by pathogens causing in tissue/organ malfunction. Recently, reports have underlined that EPC therapy has advantageous effects on sepsis models (Fan et al., 2014; Sun et al., 2020). Fan *et al.* demonstrated that CB-CD34^+^/KDR^+^ cells and SDF-1α administration synergistically improve septic animal survival through overexpression of miR-126 and -125b, key miRs in preservation of EC functionalities (Fan et al., 2014). Following, they revealed that the main EPC defensive effects on the microvascular after sepsis emergence occur through exosomes-mediated transfer of miRs like miR-126-3p and miR-126-5p (Zhou et al., 2018). Mechanistically, Zhang *et al.* suggested that EPCs mitigated sepsis-induced kidney injury by secreting miR-21-5p-containing exosomes through the silencing of RUNX1 (Zhang et al., 2021). EPC-EVs miR-126-3p and 5p suppress damage-associated molecular patterns (DAMP)-stimulated HMGB1 and vascular cell adhesion molecule 1 (VCAM1), however blockage of miR-126-3p and 5p by miR-126-3p/5p inhibitors troubled the valuable functions of EPC-exosomes. Therefore, EPC-EVs prevent unfavorable septic problems through miR-1263p/5p delivery (Zhou et al., 2018).

## 4 Pre-clinical proof of concept for CD34^+^ cell therapy

Hereby, we highlight a small portion of the numerous animal investigations that have been conducted to examine the regenerative application of CD34^+^ EPC therapy for hypoxic organ regeneration (Table 4).

**TABLE 4:** Preclinical studies of CD34^+^ cell therapy.

Authors	Condition	CD34^+^ cell source	Dosage	Delivery route	Experimental model	Outcomes	Ref
Murohara et al.	hindlimb ischemia	CB	3 × 10^6^	Intramuscular	Mouse	Increased vasculogenesis	Murohara et al. (2000a)
Madeddu et al.	hindlimb ischemia	CB	1 × 10^3^	Intramuscular	Mouse	Increased vasculogenesis and myogenesis, decrease EC apoptosis and interstitial fibrosis, enhanced limb salvage and hemodynamic recovery	Madeddu et al. (2004)
Mathiyalagan et al.	hindlimb ischemia	PB	5 × 10^6^/kg	Intramuscular	Mouse	Increased angiogenesis	Mathiyalagan et al. (2017)
Kawamoto et al.	AMI	PB	5 × 10^5^/kg	Intramyocardial	Rat	Increased vasculogenesis and cardiomyogenesis, reduced structural changes in the infarct zone, reduced inflammatory response, and improved cardiac function	Kawamoto et al. (2006)
Shalaby et al.	AMI	CB	2 × 10^6^	Intravenous	Rat	Increased angiogenesis, reduced infarct zone and the amplitude of the T-wave	Shalaby et al. (2016)
Kocher et al.	AMI	BM	2 × 10^6^	Intravenous	Rat	increased angiogenesis, reduced inflammatory response, decreased infarct size, collagen deposition, cardiomyocyte apoptosis, and improved cardiac function	Kocher et al. (2001)
Kawamoto et al.	AMI	PB	1×10^6^	Intravenous	Rat	improved regional wall motion, FS, LVEF, and myocardial neovascularization	Kawamoto et al. (2001)
Kawamoto et al.	AMI	PB	1×10^5^	Intramyocardial	Rat	increased capillary density, decreased the fibrosis rate	Kawamoto et al. (2003)
Yeh et al.	AMI	PB	2.5 × 10^6^	Intraventricular	Mouse	increased angiogenesis and cardiomyogenesis	Yeh et al. (2003)
Yoshioka et al.	AMI	BM	0.5-2 × 10^6^	Intramyocardial	Macaques	increased angiogenesis and FS	Yoshioka et al. (2005)
Taguchi et al.	cerebral ischemia	CB	5 × 10^5^	Intravenous	Mice	improved neovascularization and neurogenesis	Taguchi et al. (2004)
Shyu et al.	chronic stroke	PB	2 × 10^5^	Intracerebral	Rat	Improved vasculature, neurogenesis, and locomotor activity	Shyu et al. (2006)
Tsuji et al.	neonatal stroke	CB	1 × 10^5^	Intravenous	Mice	increased blood flow, decreased loss of ipsilateral hemispheric volume	Tsuji et al. (2014)
Chang et al.	hypoxic-ischemic injury-induced cerebral palsy	CB	1×10^5^	Intracerebral	Mice	Decreased pathological brain injury, improves neurobehavioral status	Chang et al. (2021)
Ogawa et al.	chronic stroke	PB	1×10^4^	Intracarotidal	Mice	improved neuromuscular function, spatial learning ability and escape reaction, and sensorimotor skills	Ogawa et al. (2022)

### 4.1 Hindlimb ischemia

According to studies, CB-CD34^+^ EPCs concentrated in the regenerating muscles and improved vasculogenesis and in a murine hindlimb ischemia model, leading to enhanced limb salvage and hemodynamic recovery (Murohara et al., 2000a; Schatteman et al., 2000; Madeddu et al., 2004). Collectively, CD34^+^ EPCs efficiently improve blood flow and vasculogenesis in the hindlimb ischemic models.

### 4.2 Myocardial infarction

According to data from a mouse model of experimental acute myocardial infarction (AMI), human CD34^+^ cells imbedded in the hypoxic/ischemic region and shifted toward cardiomyocytes and ECs, which related to improved heart utility (Kawamoto et al., 2006). Shalaby *et al.* compared the efficacy of human CB-CD34^+^ and CB-MSCs for the regenerate of heart tissue by stimulation of neoangiogenesis. They reported that the CD34^+^-treated group notably reduced the infarct zone and the amplitude of the T-wave and increased VEGF, Ang-1, HIF-1α, and Tie-2 levels compared to the MSC-treated subjects, suggesting the supremacy of CD34^+^ therapy vs. MSCs in the induction of regenerative pothential (Shalaby et al., 2016). Supportively, human CD34^+^ cells increased angiogenesis and reduced pro-inflammatory cytokines in the infarct zones, leading to improved cardiac function in a rat AMI model (Kim et al., 2016). Moreover, Kawamoto *et al.* found that PB-derived CD34^+^ EPCs improved regional wall motion, fractional shortening, left ventricle ejection fraction (LVEF), and myocardial neovascularization (Kawamoto et al., 2001). Also, in another study, they showed that CD34^+^ EPCs increased capillary density and decreased the fibrosis rate of the infarct area in the experimental AMI model (Kawamoto et al., 2003). Supportively, Yeh *et al.* reported that human G-CSF mobilized PB-CD34^+^ cells increased angiogenesis through migration and retention into the myocardium and trans-differentiation into ECs in ischemic tissues (Yeh et al., 2003). Furthermore, transplanting of PB-CD34^+^ cells reduce structural changes in the infarct zone and eliminates any signs of inflammatory cell recruitment seen in the transplantation of total MNCs in a rat MI model (Kawamoto et al., 2006). Also, myocardial injection of BM-CD34^+^ cells improved the vascular network and fractional shortening in the macaque MI model, supporting the efficacy of CD34^+^ cells in a large animal model of MI (Yoshioka et al., 2005). Taken together, records from preclinical studies propose that CD34^+^ cells differentiate into ECs, integrate into vascular system, and secrete angiogenic factors, promoting vascular regeneration in the microcirculation and improving myocardial perfusion in ischemia-induced tissue damage.

### 4.3 Renal failure

The effectiveness of CD34^+^ EPC therapy on the protection of CKD (chronic kidney disease) renal function has only been documented in a small number of experimental investigations. Two previous experimental studies using small and large preclinical experiments of CKD revealed that renal CD34^+^ EPC therapy considerably refurbished kidney function. In detail, Sangidorj *et al.* demonstrated that the administration of BM-CD34^+^ EPCs immunologically trafficked in the injured kidney site and postponed the collapse of kidney function aside from reduced proteinuria. As well, angiogenic molecules were increased and proinflammatory cytokines and adhesion molecules were decreased, resulting in functional and structural renal preservation in a mouse model of chronic renal failure (Sangidorj et al., 2010). In a large animal model, Chade *et al.* reported that intrarenal autologous PB-derived KDR^+^/CD34^+^ EPC injection preserved microvascular architecture and function and diminished microvascular remodeling in an experimental stenotic kidney, showing renoprotective effects of CD34^+^ EPC in a pig model of chronic renal artery stenosis (Chade et al., 2009). Also, Ohtake *et al.* reported the effectiveness of G-CSF mobilized human CD34^+^ therapy in a mice experimental model of ischemic/reperfusion acute kidney injury (AKI), which drastically upgraded renal function and repaired loss of peritubular capillaries because of ischemic condition (Ohtake et al., 2018a). Mechanistically, Vinas *et al.* reported that delivery of ECFC exosomes reduces ischemic kidney injury via transfer of miR-486-5p through targeting PTEN signaling pathway (Viñas et al., 2016). Recently, Huang *et al.* demonstrated that PB-CD34^+^ EPC therapy excellently inhibits CKD progression and kidney homeostasis deterioration by means of improvement of neoangiogenesis, blood stream, and potent anti-oxidative, anti-inflammatory, and anti-apoptosis/fibrosis capacities in a rat model (Huang et al., 2015). Taken together, the present knowledge regarding CD34^+^ therapy for kidney-related diseases supports CD34^+^ as a promising therapeutic intervention for preserving kidney functions in renovascular disease.

### 4.4 Inflammatory conditions

As above mentioned, CD34^+^ cells supply anti-inflammatory factors via paracrine activities. Comparing the cardioprotective effects of MSCs and CD34^+^ cells, Shalaby *et al.* demonstrated that CB-CD34^+^ cell therapy significantly decreased recruitment of inflammatory cells and increased angiogenic factors compared to the MSCs-treated group, which discloses the superiority of CD34^+^ therapy over MSCs in therapeutic potential (Shalaby et al., 2016). Of note, CB-derived CD133+/CD34^+^ stem/progenitors exhibit elevated amount of migratory (CXCR4)- and adhesive (LFA-1)-related antigens, allowing them to migrate and home to inflammatory areas that express higher levels of SDF-1α, a CXCR4 ligand (Das et al., 2009). Furthermore, CD34^+^ cell therapy reduces the secretion of inflammatory factors, namely TNF-α, IL-1, IL-6, and NOS2A in the lesion context while overexpression of IL-10. Moreover, human CB-CD34^+^ therapy mitigates NF-κB signaling pathway in dermal fibroblasts by increasing IL-10, providing novel mechanistic evidence of CD34^+^ cell-mediated anti-inflammatory response in a mouse model of wound healing. It worth noting that IL-10 binds to NF-κB and inhibits transcriptional function of this pathway (Kanji et al., 2014). Also, it has been shown that CD34^+^ cells enhanced epicardial and coronary microcirculation via paracrine activity–mediated angiogenic response and secretion of anti-inflammatory factors (Mackie and Losordo, 2011).

### 4.5 Cerebrovascular disease

Regarding the therapeutic effects of CD34^+^ cells in cerebral ischemia, Taguchi *et al.* have clarified that CB-derived CD34^+^ cells ameliorated angiogenesis and neurogenesis rates (Taguchi et al., 2004). Of note, injections of PB-derived CD34^+^ cells developed vasculature, neurogenesis, and locomotor activity in a chronic stroke rat experiment (Shyu et al., 2006). Supportively, Tsuji and colleagues reported that CB-CD34^+^ cells increased blood stream to the ischemic zone and decreased missing of ipsilateral hemisphere volume in a neonatal stroke model (Tsuji et al., 2014). Also, CB-CD34^+^ cells alleviates pathological brain injury and improves the neurobehavioral status in a cerebral palsy model (Chang et al., 2021). More recently, Ogawa *et al.* illustrated that transplantation of PB-CD34^+^ cells improved neurological functions, including grip strength test (to evaluate neuromuscular function), the water maze assay (to evaluate the spatial education skill), and the rotarod test (to evaluate sensorimotor skills) in a murine SCID chronic stroke model, providing another preclinical proof of the CD34^+^ therapy concept in ischemic strokes (Ogawa et al., 2022).

## 5 Clinical proof of concept for CD34^+^ cell therapy

As a result of strong preclinical background supporting CD34^+^ cells’ safety and efficacy as a treatment for injured tissues, clinical trials are now underway to use their innate abilities in the context of ischemic disorders (Leuschner et al., 2012). Here, we highlight the significant turning points of CD34^+^ cell therapy in clinical trials (Table 5).

**TABLE 5:** Clinical trials of CD34^+^ cell therapy.

Authors	Condition	CD34^+^ cell source	Dosage	Delivery route	Phase	Outcomes	Ref
Tateishi et al.	CLTI	BM	0.7−2.8 × 10^9^	Intramuscular	Pilot	Decreased pain rate and increase walking distance	Tateishi-Yuyama et al. (2002)
Kawamoto et al.	CLTI	PB	10^5^, 5 × 10^5^, and 10^6^/kg	Intramuscular	I/IIa	enhanced walking distance, pain rating, TBPI, TcPO2, decreased ulcer	Kawamoto et al. (2009)
Kinoshita et al.	CLTI	PB	10^5^, 5 × 10^5^, and 10^6^/kg	Intramuscular	I/IIa	improved TBPI and TcPO2	Kinoshita et al. (2012)
Fujita et al.	CLTI	PB	10^6^/kg	Intramuscular	II	improved pain rate, perfusion pressure, TcPO2, TBPI and pain-free walking distance	Fujita et al. (2014)
Ohtake et al.	CLTI	PB	5.44−86.8 × 10^6^	Intramuscular	II	increased the amputation-free survival index	Ohtake et al. (2018b)
Tanaka et al.	CLTI	PB	2 × 10^7^ in 20 injections	Intramuscular	I/IIa	Improved wound healing and vascular perfusion	Tanaka et al. (2014)
Losordo et al.	CLTI	PB	1 × 10^5^–1 × 10^6^/kg	Intramuscular	I/IIa	enhanced myogenesis and angiogenesis, decreased the amputation-free survival rate	Losordo et al. (2012)
Losordo et al.	CLTI	PB	1 × 10^5^–1 × 10^6^/kg	Intramuscular	I/IIa	improved quality of life and peak walking distances	Losordo et al. (2011a)
Fang et al.	CLTI	PB	10^5^, 5 × 10^5^, and 10^6^/kg	Intramuscular	—	Increased the amputation‐free survival rate, PPFWT, ulcer healing rate, and SF‐36v2 score, decreased WFPRSS and the recurrence rate	Fang et al. (2018)
Losordo et al.	Refractory angina	PB	5 × 10^4^, 1 × 10^5^, and 5 × 10^5^/kg	Intramyocardial	I/IIa	improved the frequency of angina, exercise tolerance, and CCS ranking	Losordo et al. (2007)
Losordo et al.	Refractory angina	PB	1 × 10^5^ and 5 × 10^5^/kg	Intramyocardial	II	reduced angina frequency, improved exercise tolerance, angina onset time, and CCS classification	Losordo et al. (2011b)
Henry et al.	Refractory angina	PB	1 × 10^5^ and 5 × 10^5^/kg	Intramyocardial	II	reduced angina frequency, adverse cardiac events, and mortality rate	Henry et al. (2016)
Povsic et al.	Refractory angina	PB	1 × 10^5^/kg to 1 × 10^7^	Intramyocardial	III	improved safety and function	Povsic et al. (2016)
Henry et al.	Refractory angina	PB	111 × 10^6^(x̄)	Intracoronary	Pilot	improved coronary flow reserve, CCS class, and life quality, reduced angina	Henry et al. (2022)
Corban et al.	Refractory angina	PB	1 × 10^5^/kg	Intracoronary	-	improved microvascular blood flow and SAQ scores, decreased CCS class, wilcoxon signed-rank test and nitroglycerin use	Corban et al. (2022)
Johnson et al.	Refractory angina	PB	1 × 10^4^, 1 × 10^5^, or 5 × 10^5^/kg	Intramyocardial	III	improved mortality rate, reduced cardiac-related hospital visits and expenses	Johnson et al. (2020)
Hofmann et al.	STEMI	PB	16 × 10^6^ (x̄)	Intracoronary	-	Concentration of transplanted cells in the border zone myocardium, improved cardiac regeneration	Hofmann et al. (2005)
Pasquet et al.	AMI	PB	143 × 10^6^ (x̄)	Intramyocardial	I	increased LVEF and sustained structural and functional scar repair	Pasquet et al. (2009)
Quyyumi et al.	STEMI	BM	5, 10, or 15 × 10^6^	Intracoronary	I	improved myocardial perfusion, reduced infarct size in a cell dose-dependent manner	Quyyumi et al. (2011)
Quyyumi et al.	AMI	BM	15 × 10^6^ (x̄)	Intracoronary	II	increased LVEF, decreased infarct size, hospitalized days, and mortality in a cell dose-dependent manner	Quyyumi et al. (2017)
Poglajen et al.	STEMI	PB	—	Trans-endocardial	-	antiarrhythmic benefits	Poglajen et al. (2019)
Banerjee et al.	acute ischemic stroke	PB	2.2 × 10^6^ (x̄)	Intraarterial	I	improved MRS and NIHSS scores	Banerjee et al. (2014)
Chen et al.	MCAI	PB	6.6 × 10^6^ (x̄)	Stereotaxical	II	improved the NIHSS, ESS, and the ESS motor scale	Chen et al. (2014)
Sung et al.	ischemic stroke	PB	3 × 10^7^	intra-carotid	I	increased angiogenesis, SDF-1α, the Barthel index, and CASI	Sung et al. (2018)

### 5.1 Chronic limb-threatening ischemia (CLTI)

For the first time, Tateishi *et al.* introduced the clinical utility of PB-CD34^+^ cells in an ischemic limb setting. Based on their results, autologous BM-CD34^+^ therapy ameliorate pain rate and increase walking distance 7 days after transplantation (Tateishi-Yuyama et al., 2002).

In a phase I/IIa trial of CLTI, Kawamoto and coworkers illustrated that G-CSF mobilized CD34^+^ cell transplantation enhanced pain grade, pain-free walking distance, toe brachial pressure index (TBPI), transcutaneous partial pressure of oxygen (TcPO_2_), and decreased ulcer size after 3 months of injection (Kawamoto et al., 2009); Supportively, Kinoshita *et al.* found that TBPI and TcPO_2_ parameters improved significantly 4 years after treatment, and no major amputations occurred in this period, indicating the immunity, feasibility, and efficiency of G-CSF mobilized PB-CD34^+^ cell transplantation in CLTI individuals (Kinoshita et al., 2012). Fujita *et al.* conducted a phase II clinical trial to determine endpoint selection and timing, which revealed significant improvements in perfusion pressure, pain grade, TcPO_2_, pain-free walking distance, and TBPI (Fujita et al., 2014). Ohtake *et al.* reported that G-CSF-mobilized CD34^+^ therapy increased the amputation- and CLTI-free survival index in CLTI individuals without any cell therapy-related side effects (Ohtake et al., 2018b). Tanaka and colleagues studied five dialysis individuals having non-healing diabetic feet in a phase I/IIa trial of G-CSF-mobilized CD34^+^ therapy. 18 weeks after treatment, all of the patients’ wounds had healed completely without serious adverse events (Tanaka et al., 2014). According to the initial randomized and double-blind pilot study regarding CD34^+^ therapy of CLTI, autologous G-CSF mobilized CD34^+^ therapy boosts myogenic and angiogenic activity while decreasing the amputation-free survival rate in a dose-dependent trend at 12 months (Losordo et al., 2012). This finding was later confirmed by a double-blind randomized pilot trial, which suggested that patients with severe intermittent claudication receiving autologous G-CSF mobilized CD34^+^ therapy experienced improved quality of life and longer walking distances (Losordo et al., 2011a). Fang *et al.* evaluated the long-term efficacy of G-CSF mobilized CD34^+^ cells in CLTI patients. Based on their results, CD34^+^ cells not only increased the amputation‐free survival rate, peak pain‐free walking time (PPFWT), ulcer healing rate, and SF‐36v2 score, but also decreased the Wong‐Baker faces pain rating scale and the recurrence frequency during 5years follow-up period (Fang et al., 2018).

It is worth noting that since 2017, Japan’s ministry of health (MHLW) has permitted the G-CSF-mobilized CD34^+^ cell transplantation for CLTI patients as an advanced type B medical treatment. In addition, a multicenter comparative trial of CLTI subjects aimed at guiding authorization of CD34^+^ therapy as a novel regenerative medicine-made merchandise (Identifier: NCT02501018) was launched in Japan in 2017. The MHLW designated this regenerative medicine product as an applicable pharmaceutical composition for Sakigake Strategy in 2018. The trial was finished in May 2022 with the concluding data still pending.

### 5.2 Refractory angina

Coronary microvascular dysfunction leads to angina and poor outcomes in ischemic coronary artery disease, and no specific treatment exists. As mentioned above, CD34^+^ cell transplantation expands microcirculation and improves symptoms, exercise tolerance, and mortality in preclinical studies of refractory angina patients. Nowadays, CD34^+^ transplantation has been assessed in several double-blind and randomized trials in these patients. In phase I/IIa clinical trial using the NOGATM mapping system, autologous G-CSF mobilized PB-CD34^+^ therapy improved the frequency of angina, exercise tolerance, and CCS scale, providing preliminary evidence of the immunity and bioactivity of CD34^+^ cell injection in patients with refractory angina (Losordo et al., 2007). Furthermore, in a prospective, double-blind, randomized, phase II study of refractory angina, autologous G-CSF mobilized CD34^+^ therapy considerably decreased angina frequency and upgraded exercise endurance, angina onset time, and CCS classification without any adverse cardiovascular side effects (Losordo et al., 2011b). A second year of follow-up study shows that autologous G-CSF mobilized CD34^+^ cell transplantation diminishes angina frequency, unwanted heart outcomes, and mortality rates (Henry et al., 2016). These findings are further validated in phase III of the randomized, double-blind study, which shows improved immunity and organ homeostasis in refractory angina patients undergo autologous G-CSF mobilized CD34^+^ therapy (Povsic et al., 2016). Most recently, Henry *et al.* demonstrated that autologous G-CSF mobilized CD34^+^ cells improves coronary flow reserve, CCS class, and life quality (based on the Seattle Angina Questionnaire (SAQ)) and mitigates angina frequency at 6 months after treatment (Henry et al., 2022). Supportively, Corban et al. documented that autologous G-CSF mobilized CD34^+^ therapy improved microvascular blood flow and SAQ scores and decreased CCS class, nitroglycerin use, and Wilcoxon signed-rank test at 6 months of follow-up (Corban et al., 2022). Johnson *et al.* reported that autologous G-CSF mobilized CD34^+^ therapy for refractory angina patients is associated with an improved mortality rate and reduced cardiac-related hospitalization in the year following injection compared to the year prior to transplantation (Johnson et al., 2020). In a one more general note, it could be said that CD34^+^ therapy is immune and could be a potent operative regenerative approach for microvascular dysfunction and angina patients.

### 5.3 Myocardial Infarction

According to Hofmann *et al.* injected CD34^+^ cells may effectively accumulate in the area of ischemia and improve cardiac regeneration, While a significant portion of the injected-MNCs cramped in the spleen, liver, and the infarcted myocardium’s center, providing another proof of the superiority of CD34^+^ cells over MNCs (Hofmann et al., 2005). In 2009, Pasquet *et al.* launched a pilot study using autologous G-CSF-mobilized PB-CD34^+^ cells to treat AMI subjects, which resulted in increased LVEF and sustained structural/functional scar repair (Pasquet et al., 2009). In a phase I dose-dependent investigation of subjects with LVEF≤ 50% after angioplasty for STEMI, Quyyumi et al. found that BM-CD34^+^ cells improved myocardial perfusion and reduced infarct size in a dose-dependent manner (Quyyumi et al., 2011). The PreSERVE-AMI was the largest randomized phase II trial that used autologous BM-CD34^+^ cell transplantation of LV dysfunction post-STEMI. After 12 months, transplanted subjects showed decreased infarct size, hospitalized days, and mortality compared to the non-treated group and increased LVEF in a dose-dependent manner, providing additional proof of the immunity and efficiency of CD34^+^ therapy in STEMI subjects (Quyyumi et al., 2017). Besides, Poglajen *et al.* demonstrated potential antiarrhythmic benefits of autologous G-CSF mobilized CD34^+^ cells on ventricular arrhythmias subjects (Poglajen et al., 2019). These findings further supported the immunity and efficiency of CD34^+^ cell transplantation in STEMI-suffered individuals.

### 5.4 Cerebrovascular disease

According to the first report of using autologous G-CSF mobilized CD34^+^ therapy in acute ischemic stroke patients, CD34^+^ cells improved modified rankin score (MRS) and national institutes of health stroke score (NIHSS) in the 6-month follow-up (Banerjee et al., 2014). Similarly, Chen *et al.* showed in a randomized, single-blind, controlled trial that autologous G-CSF mobilized CD34^+^ therapy improved the NIHSS, the European stroke score (ESS), and the ESS motor score without any serious adverse events in patients with a MCAI (Chen et al., 2014). Furthermore, Sung *et al.* reported that autologous G-CSF mobilized PB-CD34^+^ cell transplantation increased angiogenesis, the Barthel index, and cognitive ability screening instrument score (CASI) at 6-month follow-up after transplantation without any recurrent stroke or tumorigenesis (Sung et al., 2018). Based on these findings, a randomized double-blind study of CD34^+^ therapy in chronic ischemic stroke (Identifier: jRCT2052200112) began in 2020 and is still recruiting subjects.

### 5.5 Renal failure

Regarding CD34^+^ clinical trials of kidney disease, in a randomized controlled study, Yang *et al.* recently found that intra-renal transplantation of autologous G-CSF mobilized CD34^+^ cells for CKD patients significantly reduced adverse clinical outcomes (dialysis and death) at 1 year compared to the control group, but did not improve kidney function (Yang et al., 2020). Furthermore, according to Lee *et al.*, G-CSF-mobilized CD34^+^ transplantation into the right renal artery is 100% safe and maintains the renal function in a stationary state, suggesting that CD34^+^ transplantation could mitigate the collapse of renal homeostasis in CKD subjects (Lee et al., 2017). Besides, in phase I/II study, Suzuki and colleagues reported that direct injection of G-CSF-mobilized CD34^+^ cells to both renal arteries of a 36 years old AKI patient significantly improved the serum creatinine level, GFR, angiogenic-related cytokines, and consequently kidney function 23 weeks after cell therapy without major adverse events (Suzuki et al., 2021). Therefore, CD34^+^ cell transplantation could apply renoprotective effects by improved angiogenesis and anti-inflammation capability and could be considered a new therapeutic tool for kidney disorders.

## 6 Conclusion

Since the discovery of CD34^+^ population in PB as the EPC-fortified fraction in 1997, a growing body of preclinical and clinical studies using CD34^+^ cells, a naturally occurring microvascular repair cell, provide undeniable evidence for the immunity and efficiency of CD34^+^ EPC treatment. In fact, until now, there has been no unsuccessful CD34^+^ cell therapy studies for tissue regeneration that has failed to provide a piece of evidence for safety and clinical effect. Remarkably, these reports were carried out on patients who were at the end of their treatment options. Despite being given to these seriously ill patients, a single CD34^+^ cell therapy administration has produced convincing proof that ischemia can be reversed. Principally, a multicenter trial in CLTI-suffered individuals was initiated in 2017in Japan with the aim of guiding authorization of CD34^+^ therapy as a novel regenerative medicine-made merchandise, which has been nominated as an applicable approach for the Sakigake Strategy. In case of therapeutically approval of this product from now on, it is anticipated to be a breakthrough for developing signals for diseases aside from CLTI. Furthermore, in the United States, the FDA has designated CD34^+^ cell products as “regenerative medicine advanced therapy” for refractory angina. If these studies are completed, approved, and expanded successfully, CD34^+^ therapy may result in the world’s first approval of a regenerative therapeutic approach for CVD and other vascular-related diseases.
